# Endocrine disrupting chemical-associated hair product use during pregnancy and gestational age at delivery: a pilot study

**DOI:** 10.1186/s12940-021-00772-5

**Published:** 2021-07-28

**Authors:** Emma V. Preston, Victoria Fruh, Marlee R. Quinn, Michele R. Hacker, Blair J. Wylie, Karen O’Brien, Shruthi Mahalingaiah, Tamarra James-Todd

**Affiliations:** 1 Department of Environmental Health, Harvard T.H. Chan School of Public Health, Boston, MA 02115 USA; 2 Department of Obstetrics and Gynecology, Beth Israel Deaconess Medical Center, Harvard Medical School, Boston, MA 02115 USA; 3 Department of Obstetrics and Gynecology, Division of Reproductive Endocrinology and Infertility, Massachusetts General Hospital, Boston, MA 02114 USA; 4 Department of Epidemiology, Harvard T.H. Chan School of Public Health, Boston, MA 02115 USA

**Keywords:** Preterm birth, Hair products, Personal care products, Endocrine disrupting chemicals, Pregnancy

## Abstract

**Background:**

Prenatal endocrine disrupting chemical (EDC) exposure has been associated with increased risk of preterm birth. Non-Hispanic Black women have higher incidence of preterm birth compared to other racial/ethnic groups and may be disproportionately exposed to EDCs through EDC-containing hair products. However, research on the use of EDC-associated hair products during pregnancy and risk of preterm birth is lacking. Therefore, the objective of this pilot study was to estimate associations of prenatal hair product use with gestational age at delivery in a Boston, Massachusetts area pregnancy cohort.

**Methods:**

The study population consisted of a subset of participants enrolled in the Environmental Reproductive and Glucose Outcomes (ERGO) Study between 2018 and 2020. We collected self-reported data on demographics and hair product use using a previously validated questionnaire at four prenatal visits (median: 12, 19, 26, 36 weeks’ gestation) and abstracted gestational age at delivery from medical records. We compared gestational age and hair product use by race/ethnicity and used linear regression to estimate covariate-adjusted associations of product use and frequency of use at each study visit with gestational age at delivery. Primary models were adjusted for maternal age at enrollment and delivery method.

**Results:**

Of the 154 study participants, 7% delivered preterm. Non-Hispanic Black participants had lower mean gestational age at delivery compared to non-Hispanic White participants (38.2 vs. 39.2 weeks) and were more likely to report ever and more frequent use of hair products. In regression models, participants reporting daily use of hair oils at visit 4 had lower mean gestational age at delivery compared to non-users (β: -8.3 days; 95% confidence interval: -14.9, -1.6). We did not find evidence of associations at earlier visits or with other products.

**Conclusions:**

Frequent use of hair oils during late pregnancy may be associated with shorter gestational duration. As hair oils are more commonly used by non-Hispanic Black women and represent potentially modifiable EDC exposure sources, this may have important implications for the known racial disparity in preterm birth.

**Supplementary Information:**

The online version contains supplementary material available at 10.1186/s12940-021-00772-5.

## Background

Preterm birth is defined as birth before 37 weeks of gestation and affects an estimated 10% of pregnancies in the United States [1]. Preterm birth is associated with significant neonatal mortality and morbidity as well as adverse health outcomes throughout childhood and later life, such as neurodevelopmental delays, cardiometabolic disease, allergy, and asthma**,** representing a major economic and societal burden [2–5]. While rates of preterm birth in the United States have fluctuated over past decades, there is a marked and increasing racial disparity in the incidence of preterm birth, with non-Hispanic Black women having greater than 50% increased risk of preterm birth compared with non-Hispanic White women (14% vs. 9% in 2018) [1, 2, 4, 6]. Both the etiology of preterm birth and the underlying factors contributing to the racial/ethnic disparity are poorly understood, and the disparity cannot be explained by differences in sociodemographic factors alone [4]. Therefore, it is critical to identify modifiable risk factors, such as exposure to endocrine disrupting chemicals (EDCs), which may contribute to preterm birth risk and the marked racial disparity.

Exposure to EDCs, including phthalates and parabens, has been associated with increased risk of preterm birth [3, 6–10]. Racial/ethnic differences in exposure to these chemicals have been observed, with non-Hispanic Black pregnant and non-pregnant women having higher concentrations of some EDCs, including certain phthalates and parabens, compared to non-Hispanic White women in the U.S. [11–14]. These EDCs are commonly found in personal care products, including hair products, resulting in widespread personal exposure through dermal absorption and inhalation [15]. In particular, hair products marketed towards and more commonly used by non-Hispanic Black women have been shown to contain multiple EDCs, including parabens, phthalates, and phenols [15, 16], and recent evidence suggests that these products may be hormonally active [17, 18]. Furthermore, the use of certain hair products has been associated with multiple hormonally-mediated health outcomes, such as earlier age at menarche, breast cancer, and uterine leiomyomata [19–27], indicating that differences in hair product use patterns could potentially contribute to the observed disparities in EDC exposure and women’s reproductive health outcomes [13, 28]. However, research on the use of EDC-associated hair products during pregnancy and risk of preterm birth or earlier gestational age at delivery is lacking.

Therefore, the objective of this study was to examine the association of hair product use during pregnancy with gestational age at delivery in a subset of participants from a Boston, Massachusetts (MA) area pregnancy cohort. We hypothesized that the use of EDC-associated hair products and more frequent use during pregnancy would be associated with earlier gestational age at delivery.

## Methods

### Study population

The study population consists of a subset of the Environmental Reproductive and Glucose Outcomes (ERGO) Study enrolled at Beth Israel Deaconess Medical Center (BIDMC) in Boston, MA. Pregnant participants were recruited during routine prenatal visits in early pregnancy (median 13 weeks) beginning in 2018. Participants were eligible if they were ≥ 18 years old, < 15 weeks of gestation, had plans to receive prenatal care and deliver at BIDMC, and spoke English. Participants carrying triplets or higher order multiples or with preexisting Type 1 or Type 2 diabetes, and those with the inability to tolerate an oral glucose tolerance test were ineligible. After enrollment, participants were followed through delivery and into the early postpartum period. Of the 193 participants enrolled at BIDMC, 154 had delivered at the time of this analysis (September 2020) and had data on both gestational age at delivery and at least one hair product type from one or more prenatal study visits.

Study protocols were approved by the institutional review boards of all participating institutions and all participants provided written informed consent.

#### Hair product use

Data on hair product use were collected from participant questionnaires at up to four prenatal visits. The median gestational ages at visits 1 through 4 were: 12, 19, 26, and 36 weeks. We included hair product use data from all available participant questionnaires. For example, for participants who delivered prior to completing visit 4, we included all available data from visits 1–3.

We assessed hair product use via a validated questionnaire, which has been previously used in exposure assessment and epidemiologic studies [15, 16, 18, 29]. The questionnaire queried the use of 8 categories of hair products during the month prior to the prenatal visit (Yes/No) and the frequency of use (“Daily”, “Once a week or more”, “Once a month or more”, “Every 6 months or more”, “Other”). Participants were asked about their use of the following hair product categories with example products for each category: hair oils, hair lotion, leave-in conditioners, creams, or hair mayonnaise (leave-in conditioners), non-lye perms/relaxers, lye-perms/relaxers, hair care product prescribed by a doctor (prescription products), natural hair products, and other products.

We assessed hair product use in four ways: (1) ever/never use during pregnancy, where ever use was defined as positive report of product use within the last month on any questionnaire, (2) use within the month prior to each prenatal visit, (3) daily vs. less than daily use within the last month, at each time point, and (4) total product categories used during pregnancy. Due to missing data, frequency of use data were only available for a subset of participants depending on the hair product type and visit number, ranging from *n* = 126 for leave-in conditioners at visit 4 to *n* = 140 for multiple products at visit 2.

#### Outcome assessment

Gestational age at delivery (weeks) was obtained from electronic medical records and was modeled continuously for all analyses. At BIDMC, gestational age is estimated as a best obstetric estimate using the individual’s self-reported last menstrual period and confirmed with the earliest ultrasound obtained in accordance with criteria established by the American College of Obstetrics and Gynecology, the Society for Maternal–Fetal Medicine, and the American Institute of Ultrasound in Medicine [30]. Preterm birth was defined as birth prior to 37 weeks of completed gestation.

#### Covariate assessment

We collected self-reported data on the following maternal sociodemographic characteristics from questionnaires at the first prenatal visit: pre-pregnancy body mass index (BMI, kg/m^2^), insurance status (private vs. other), education (bachelor’s degree or higher vs. no bachelor’s degree), and race/ethnicity (categorized as non-Hispanic Black, non-Hispanic White, or Other for our analyses). Due to small numbers, the Other category included participants who self-reported race/ethnicity as non-Hispanic Haitian/Caribbean, Native Hawaiian or other Pacific Islander, Asian, American Indian/Alaskan Native, Hispanic, or more than one race. Maternal date of birth and delivery method were obtained from electronic medical records. We did not have data on whether deliveries were preceded by spontaneous onset of labor. However, we used delivery method (cesarean delivery, spontaneous vaginal, induced vaginal) as a proxy measure for spontaneous versus medically indicated delivery.

### Statistical analysis

We assessed the proportion and frequency of use of individual hair product types at each prenatal visit in all participants and by race/ethnicity. We assessed differences in mean gestational age at delivery by race/ethnicity using an F-statistic and *p*-value. We used linear regression to assess associations of hair product use and frequency during pregnancy and at each visit with continuous gestational age at delivery, fitting separate models for each product category. We were only able to evaluate associations for hair oils, hair lotions, and leave-in conditioners, as there was low use of other product categories and insufficient power to model their associations with gestational age at delivery.

Based on prior evidence, we considered the following covariates as potential confounders of the association between hair product use and gestational age at delivery: maternal age at enrollment (years), race/ethnicity, and educational attainment (bachelor’s degree or higher vs. no bachelor’s degree). Due to our small sample size and limited power, we added each additional covariate to our regression models individually and those covariates that did not meaningfully change our results were not included in our final parsimonious models [31]. As delivery method is strongly associated with gestational age, we adjusted for delivery method in our models to reduce variability in our outcome measure. Our final models included maternal age at enrollment and delivery method. In secondary models, we additionally adjusted for maternal race/ethnicity. However, we had sparse frequency of use data by race/ethnicity and results from the race/ethnicity adjusted models should be interpreted with caution.

As a sensitivity analysis, we restricted our regression models to participants who had spontaneous vaginal deliveries. Additionally, due to the potential change in hair product use patterns after COVID-19 restrictions, we conducted a sensitivity analysis restricted to participants who delivered prior to March 1^st^, before COVID-19 restrictions were enacted in Massachusetts. We used SAS version 9.4 (SAS, Cary, NC) for all statistical analyses.

## Results

Among the 154 participants included in our analytic cohort from 2018 to 2020, mean (standard deviation, SD) maternal age at enrollment was 32.4 (4.5) years, BMI at the first visit was 25.6 (5.4) kg/m^2^, and gestational age at delivery was 39.1 (1.9) weeks (Table 1). Twenty-four percent of participants delivered via cesarean and most participants had a bachelor’s degree or higher (82%). Fifty percent of participants were non-Hispanic White and 8% were non-Hispanic Black, with 42% self-reporting another race/ethnicity (Table 1). Mean (SD) gestational age at delivery was 38.2 (2.8) weeks for non-Hispanic Black participants, 39.2 (2.2) weeks for non-Hispanic White participants, and 39.3 (1.3) weeks for participants in the Other racial/ethnic category. The proportion of participants delivering preterm was higher among non-Hispanic Black participants (17%) than among non-Hispanic White participants (8%) or participants of Other race/ethnicity (3%). The proportion of participants with spontaneous vaginal deliveries was higher among non-Hispanic Black participants (67%) compared to non-Hispanic White participants (43%) or participants of Other race/ethnicity (55%) (Supplemental Table S1). When assessing differences in gestational age by race/ethnicity, mean gestational age at delivery was approximately 6 days earlier in non-Hispanic Black participants compared to non-Hispanic White participants (β: -6.6 days; 95% Confidence Interval (CI): -14.9, 1.6), but was not noticeably different for participants of Other race/ethnicity (β: 0.9 days; 95% CI: -3.6, 5.4) compared to non-Hispanic White participants. The most commonly ever used hair products among participants in our cohort were oils (36%), lotions (33%), and leave-in conditioners (41%). Less than 4% of participants reported ever use of either lye or non-lye perms/relaxers (Table 2). A higher proportion of non-Hispanic Black participants reported ever use of hair oils (92%), hair lotions (83%), and leave-in conditioners (83%) compared to non-Hispanic White participants (26%, 18%, and 35%, respectively, Table 2). The mean number of product categories used during pregnancy among all participants was 1.7 product categories. Participants who reported using hair oils during pregnancy used a greater number of hair product categories compared to non-users during pregnancy (mean: 3.39 vs. 0.66 product categories; *p* < 0.0001).

**Table 1:** Participant Characteristics (*n* = 154)

Characteristic	n	Mean ± SD or %^a^	Range
Age at enrollment (years)	154	32.4 ± 4.5	20.0 − 45.0
Body mass index at 1st visit (kg/m^2^)	153	25.6 ± 5.4	17.5–47.7
Gestational age at delivery (weeks)	154	39.1 ± 1.9	24.1 − 41.7
Preterm birth (< 37 weeks)	10	7%	
Delivery method	154		
Cesarean	37	24%	
Vaginal—induced	40	26%	
Vaginal—spontaneous	77	50%	
Education	141		
Bachelor’s degree or higher	116	82%	
No bachelor’s degree	25	18%	
Race/ethnicity	154		
Non-Hispanic Black	12	8%	
Non-Hispanic White	77	50%	
Other	65	42%	

^a^Percentages may sum to more than 100% due to rounding

**Table 2:** Hair Product Use by Race/Ethnicity^a^ (*n* = 154)

			Race/Ethnicity^b^			
Product	Timepoint		All	Non-Hispanic White	Non-Hispanic Black	Other race/ethnicity
		n	n (%)	n (%)	n (%)	n (%)
Hair oils	Ever	154	56 (36.4)	20 (26.0)	11 (91.7)	25 (38.5)
	Visit 1	139	30 (21.6)	10 (13.9)	6 (85.7)	14 (23.3)
	Visit 2	140	33 (23.6)	11 (15.3)	7 (63.6)	15 (26.3)
	Visit 3	139	30 (21.6)	11 (15.3)	8 (80)	11 (19.3)
	Visit 4	129	26 (20.2)	6 (8.8)	5 (83.3)	15 (27.3)
Hair lotion	Ever	154	51 (33.1)	14 (18.2)	10 (83.3)	27 (41.5)
	Visit 1	139	24 (17.3)	7 (9.7)	4 (57.1)	13 (21.7)
	Visit 2	139	30 (21.6)	9 (12.7)	6 (54.6)	15 (26.3)
	Visit 3	138	25 (18.1)	5 (7.0)	7 (70.0)	13 (22.8)
	Visit 4	129	17 (13.2)	4 (5.9)	5 (83.3)	8 (14.6)
Leave-in conditioners	Ever	154	63 (40.9)	27 (35.1)	10 (83.3)	26 (40.0)
	Visit 1	138	31 (22.5)	15 (20.8)	3 (42.9)	13 (22.0)
	Visit 2	139	33 (23.7)	14 (19.7)	7 (63.6)	12 (21.1)
	Visit 3	139	28 (20.1)	11 (15.3	8 (80.0)	9 (15.8)
	Visit 4	126	23 (17.8)	8 (11.8)	3 (50.0)	12 (21.8)
Non-lye perms/relaxers	Ever	154	5 (3.6)	0 (0)	1 (8.3)	4 (6.2)
	Visit 1	139	2 (1.4)	0 (0)	1 (14.3)	1 (1.7)
	Visit 2	139	3 (2.2)	1 (0)	2 (33.3)	3 (66.7)
	Visit 3	139	2 (1.4)	0 (0)	1 (14.3)	1 (1.7)
	Visit 4	128	1 (0.8)	0 (0)	1 (16.7)	0 (0)
Lye perms/relaxers	Ever	154	3 (2.0)	1 (3.3)	0 (0)	2 (3.1)
	Visit 1	138	0 (0)	0 (0)	0 (0)	0 (0)
	Visit 2	140	3 (2.1)	1 (1.4)	0 (0)	2 (3.5)
	Visit 3	139	0 (0)	0 (0)	0 (0)	0 (0)
	Visit 4	129	1 (0.8)	0 (0)	0 (0)	1 (1.8)
Natural	Ever	154	39 (25.3)	7 (9.1)	9 (75.0)	23 (35.4)
	Visit 1	138	19 (13.8)	1 (1.4)	2 (28.6)	16 (27.1)
	Visit 2	139	21 (15.1)	3 (4.2)	6 (54.6)	12 (21.1)
	Visit 3	139	17 (12.2)	3 (4.2)	5 (50.0)	9 (15.8)
	Visit 4	127	15 (11.8)	3 (4.6)	2 (33.3)	10 (18.2)
Prescription	Ever	153	9 (5.9)	1 (1.3)	4 (33.3)	4 (6.3)
	Visit 1	137	1 (0.7)	0 (0)	0 (0)	1 (1.7)
	Visit 2	138	8 (5.8)	1 (1.4)	4 (36.4)	3 (5.5)
	Visit 3	139	2 (1.4)	1 (1.4)	1 (10)	0 (0)
	Visit 4	129	3 (2.3)	0 (0)	1 (16.7)	2 (3.6)
Other	Ever	153	29 (19.0)	12 (15.6)	7 (58.3)	10 (15.6)
	Visit 1	139	8 (5.8)	3 (4.2)	1 (14.3)	4 (6.7)
	Visit 2	136	13 (9.6)	4 (5.6)	4 (36.4)	5 (9.3)
	Visit 3	139	10 (7.2)	4 (5.6)	4 (40.0)	2 (3.5)
	Visit 4	128	9 (7.0)	3 (4.5)	2 (33.3)	4 (7.3)

^a^Defined as use within the last month

^b^Percent for ‘All’ is percentage of total n for each visit; Percent for non-Hispanic White, non-Hispanic Black or Other race/ethnicity is percentage of total n within each race/ethnicity category at each visit

### Hair product use & gestational age at delivery

In regression models evaluating hair product use, daily use of hair oils at visit 4 was associated with earlier mean gestational age at delivery compared to non-use (β: -7.9 days; 95% CI: -14.3, -1.5), adjusting for maternal age at and delivery method (Table 3, Model A). Additional adjustment for race/ethnicity did not appreciably change effect estimates (Table 3, Model B; β: -8.3 days; 95% CI: -14.9, -1.6) and the effect of race/ethnicity on gestational age at delivery was not significant in the multivariable models. Additionally, in a sensitivity analyses we found that the association between daily hair oil use at visit 4 and gestational age at delivery was slightly stronger (β: -9.0 days; 95% CI: -15.9, -2.2) when restricting to non-Hispanic White and Other race participants. Results for other products were similar, with participants reporting daily use of hair lotions or leave-in conditioners at visit 4 had an earlier mean gestational age at delivery compared to non-users, though the magnitude of the associations was not as strong and point estimates were imprecise for these models [(β: -2.1 days; 95% CI: -12.4, 8.2) and (β: -2.2 days; 95% CI: -9.6, 5.2), respectively]. Results were similar in unadjusted models (Supplemental Table S2). We observed little evidence of earlier gestational age at delivery with other types of hair products, less frequent product use (< daily, never), and for hair product use earlier in pregnancy (visits 1–3). In sensitivity analyses, restricting to participants with spontaneous vaginal deliveries did not meaningfully alter the association between daily hair oil use at visit 4 and lower gestational age at delivery (β: -7.9 days; 95% CI: -14.3, -1.5) (Supplemental Table S3). Similarly, when we restricted our analyses to participants who delivered prior to March 1^st^, 2020, daily use of hair oils at visit 4 remained associated with lower mean gestational age at delivery compared to non-users, with a suggestively stronger association (β: -9.7 days; 95% CI: -16.5, -2.8) (Supplemental Table S4). The percentage of never users of hair oils was similar for participants who delivered prior to March 1^st^, 2020 (76–80%) compared to those who delivered on March 1^st^ 2020 or after (78–82%) across visits. Daily hair oil use was more consistent across visits for participants who delivered prior to March 1^st^, 2020 (7–10%) compared to those who delivered on or after March 1^st^, 2020 (0–12%).

**Table 3:** Covariate-Adjusted Differences in Mean Gestational Age (Days) at Delivery Associated with Frequency of Hair Product Use During Pregnancy (*n* = 154)

			Hair Oil			Hair Lotion			Leave-In Conditioners	
Model Timepoint			Model A^a^	Model B^b^		Model A^a^	Model B^b^		Model A^a^	Model B^b^
	Frequency	n	β (95% CI)		n	β (95% CI)		n	β (95% CI)	
Visit 1		134			136			134		
	Daily	7	-4.9 (-15.1, 5.4)	-5.8 (-16.3, 4.7)	4	3.9 (-9.7, 17.4)	3.1 (-10.7, 16.9)	7	5.1 (-5.3, 15.4)	5.1 (-5.3, 15.6)
	< Daily	18	1.0 (-5.7, 7.6)	0.5 (-6.7, 7.7)	17	-3.2 (-10.0, 3.5)	-3.8 (-10.7, 3.2)	20	1.7 (-4.7, 8.1)	1.7 (-4.8, 8.2)
	Never	109	0 (ref)	0 (ref)	115	0 (ref)	0 (ref)	107	0 (ref)	0 (ref)
Visit 2		136			136			137		
	Daily	10	0.04 (-8.7, 8.8)	-0.1 (-9.2, 9.0)	10	-0.8 (-9.7, 8.0)	-1.1 (-10.3, 8.0)	6	-5.0 (-12.9, 2.8)	-4.1 (-12.2, 4.0)
	< Daily	19	-2.3 (-8.9, 4.3)	-2.2 (-8.9, 4.6)	17	-0.7 (-7.6, 6.2)	-0.7 (-7.7, 6.3)	20	-0.4 (-4.5, 3.8)	0.0 (-4.2, 4.3)
	Never	107	0 (ref)	0 (ref)	109	0 (ref)	0 (ref)	106	0 (ref)	0 (ref)
Visit 3		135			134			136		
	Daily	14	-1.1 (-6.4, 4.2)	-1.0 (-6.7, 4.6)	9	1.4 (-5.3, 8.0)	1.7 (-5.4, 8.7)	5	-2.4 (-11.0, 6.2)	-2.5 (-11.5, 6.5)
	< Daily	12	-1.3 (-6.9, 4.3)	-1.3 (-7.0, 4.5)	12	-2.4 (-8.0, 3.1)	-2.3 (-8.0, 3.5)	20	-2.4 (-6.9, 2.0)	-2.5 (-7.1, 2.2)
	Never	109	0 (ref)	0 (ref)	113	0 (ref)	0 (ref)	111	0 (ref)	0 (ref)
Visit 4		129			127			129		
	Daily	8	-7.9 (-14.3, -1.5)	-8.3 (-14.9, -1.6)	3	-2.1 (-12.4, 8.2)	-1.8 (-12.6, 9.0)	6	-2.2 (-9.6, 5.2)	-2.1 (-9.6, 5.4)
	< Daily	18	0.9 (-3.5, 5.3)	0.9 (-3.8, 5.7)	12	-2.8 (-8.2, 2.6)	-3.0 (-9.0, 3.0)	17	-0.4 (-5.0, 4.2)	-0.4 (-5.0, 4.3)
	Never	103	0 (ref)	0 (ref)	112	0 (ref)	0 (ref)	106	0 (ref)	0 (ref)

^a^Adjusted for maternal age at enrollment (years) and delivery method (cesarean, induced vaginal, spontaneous vaginal)

^b^Additionally adjusted for maternal race/ethnicity (non-Hispanic Black, non-Hispanic White, Other race/ethnicity)

In regression models of ever versus never product use during pregnancy, we did not find evidence of associations between ever use of hair oils (β: -1.7 days; 95% CI: -6.2, 2.8), hair lotions (β: -1.7 days; 95% CI: -6.4, 2.9), or leave-in conditioners (β: -0.4 days; 95% CI: -4.8, 4.0) with mean gestational age at delivery, adjusting for maternal age and delivery method. Total product categories used was not associated with gestational age at delivery (β: -0.6 days; 95% CI: -1.8, 0.6), adjusting for maternal age and delivery method.

## Discussion

In this pilot study of a subset of pregnant participants from the ERGO cohort, we observed an association between daily use of hair oils in late pregnancy (median of 36 weeks of gestation) and earlier gestational age at delivery when compared to non-users. Although effects were not as strong for hair lotion and leave-in conditioners, we observed a trend of shorter gestation for daily users when compared with non-users at the fourth prenatal visit for these products. Overall, we did not find evidence of earlier gestational age at delivery for other types of hair products or less than daily use of hair products. Within a subset of participants who delivered prior to COVID-19 restrictions, we found a slightly stronger association between daily use of hair oils in late pregnancy and earlier gestational age at delivery when compared to non-users.

Previous literature on the use of EDC-associated hair products and risk of adverse birth outcomes is limited, and it is challenging to compare findings across studies due to the differences in the specific hair products and time periods being evaluated. The majority of previous studies have focused on hair dyes and relaxers, which likely have significantly different chemical profiles and patterns of use compared to hair oils. For example, hair oils are typically used more frequently and are not rinsed out as quickly compared to dyes and relaxers [15]. In a nested case–control study of Chinese mother-infant pairs (*n* = 210 controls, *n* = 105 cases), use of hair dyes prior to conception was associated with greater odds of delivering a low-birth weight infant (odds ratio (OR): 1.71; 95% CI: 1.01, 2.92) [32]. However, data on dye use during pregnancy were not collected. One case–control study (*n* = 304 controls; *n* = 188 preterm and *n* = 156 low birth weight cases) among Black women in the United States evaluated self-reported chemical curl/straightening hair product use during pregnancy or within the three months prior to conception, finding no evidence that users of either hair straightener products or curl products had an increased risk of preterm birth (adjusted OR: 0.70; 95% CI: 0.4, 1.1; adjusted OR: 0.90, 95% CI: 0.50, 1.80, respectively) or low birth weight (adjusted OR: 0.60; 95% CI: 0.4, 1.1; adjusted OR: 1.00, 95% CI: 0.50, 1.90, respectively) compared to those unexposed [33]. Furthermore, a case–control analysis among Black women from the Black Women’s Health Study (*n* = 5,633 controls; *n* = 497 cases) observed no association between self-reported ever use of hair relaxers and preterm birth when compared to never use (adjusted OR: 1.00, 95% CI: 0.60,1.80) [34]. Our current work addresses some constraints of previous studies by assessing several types of hair products (e.g., hair oils, hair lotion, and leave-in conditioners) and by evaluating the associations between use of these hair product types during pregnancy and gestational age at delivery prospectively.

Our evaluation of hair product use and gestational age at delivery by race/ethnicity within this study relates to prior work describing racial disparities in preterm birth rates [1, 4–6] and differences in hair product use patterns between non-Hispanic Black and non-Hispanic White women [15, 16, 35]. Eighty-three to 92% of non-Hispanic Black participants in our analysis reported ever use of hair oils, lotions, or leave-in conditioners compared to 18–35% of non-Hispanic White participants. Gestational age at delivery was earlier for non-Hispanic Black participants (mean: 38.2; median: 39.1 weeks) compared to non-Hispanic White participants (mean: 39.2; median: 39.4 weeks). However, due to the limited number of non-Hispanic Black participants within our study, we were unable to evaluate associations stratified by race/ethnicity. Future studies in larger, more diverse cohorts are needed to determine whether potentially modifiable risk factors, such as hair product use, contribute to racial disparities in preterm birth risk.

Many frequently used hair products contain EDCs such as phthalates and parabens. One hypothetical pathway that may explain observed associations of shorter gestation with more frequent use of hair oils is the presence of phthalates. In fact, previous work has shown that phthalates are present, and likely included as fragrance, in commonly used hair products [15, 16]. Phthalates may alter inflammatory pathways associated with preterm parturition [36–38]. Preterm birth as a potential result of inflammation has been described in previous work [39, 40], and was proposed as a potential mechanism for observed associations between phthalate metabolites and increased odds of preterm birth [3, 41]. Parabens may additionally impact estrogen and progesterone receptor signaling [42, 43], potentially resulting in abnormal placental development contributing to preterm delivery [44, 45].

Our analysis suggests stronger associations for daily hair oil use during the month prior to our last study visit during pregnancy (visit 4: median 36 weeks). The presence of phthalates in hair oils may explain some of the observed associations with shorter gestation. Previous work has investigated potential prenatal windows of susceptibility for the effect of phthalate exposure on gestational length [35, 40]. For example, prior studies have observed stronger associations for preterm birth with urinary phthalate metabolites measured at a median of 26 weeks of gestation (vs. 10, 18 or 35 weeks) [46] and 23 weeks of gestation (vs. 18 or 28 weeks) [41]. While these studies observed the strongest associations at earlier points in mid- to late-pregnancy compared to our visit 4 (median 36 weeks), it is important to remember that our data represents daily use in the month prior to the study visit. As phthalates may alter inflammatory pathways associated with parturition [36–38], it is possible that exposures occurring during mid- to late-pregnancy may accelerate this process. Still, this previous work was explicitly evaluating phthalate metabolites rather than exposure to hair products, which likely contain a mixture of EDCs (e.g., phthalates, parabens) that may mutually impact potential windows of susceptibility for birth outcomes. Further studies are needed to explore potential sensitive windows during pregnancy to the effects of EDCs and EDC-associated hair products on gestational duration.

This study has several limitations. First, there is potential for misclassification of hair product use due to self-reported data. Individuals may have had difficulty differentiating products used and their appropriate category. However, our study and previous work have shown hair oils to be associated with adverse female reproductive health outcomes [29, 47] and to be hormonally active [18]. Second, we lacked information on the specific hair product brand names used by each participant, the number of products used within each category, the total amount of product used/applied, and the EDC formulation for each product; therefore, we were unable to assess factors that likely created significant variation in individuals’ product-related EDC exposure. Although we did not obtain information on duration of use prior to pregnancy, we collected data on product use as well as frequency of use within the month prior to each prenatal visit. Third, as this work was part of a relatively small pilot study, we had difficulty with sparse data across frequency of use categories, which may have reduced our ability to detect associations between product use frequency and gestational age at delivery for some products. Collapsing use frequency categories into daily, less than daily, or non-users may not have captured the relevant usage patterns for all products, such as those used only occasionally. Additionally, while we were able to adjust our models for maternal age, delivery method, and race/ethnicity, we had insufficient power to adjust our regression models for a wider range of potential confounding factors. That said, to our knowledge, few factors are associated with both hair product use and gestational age, and therefore few additional confounding factors may exist when considering this association. Fourth, we did not assess use of other personal care products in this analysis. It is possible that hair oil use is a proxy for other product use, exposures, or behaviors that are associated with decreased gestational age at delivery. Fifth, we had a limited number of participants as previously noted with relatively low reported product use. Therefore, our findings should be confirmed in a larger and more diverse cohort with varied frequency of use data for multiple hair products. Sixth, we evaluated gestational age as opposed to preterm birth. Additionally, participants in our cohort with preterm deliveries prior to completing the visit 4 questionnaire (*n* = 5) were not captured in the visit 4 analysis and we therefore may not have captured the most relevant exposure window for all participants. Finally, due to the small sample size, we were unable to assess specific delivery indications (e.g., fetal distress, maternal disorder) beyond adjusting for delivery method. Including the reasons for earlier delivery in future studies could shed further light on specific risks of EDC-associated product use on pregnancy complications.

Despite these limitations, this study had several strengths. To our knowledge, our study is the first prospective study to evaluate hair oil, lotion, and leave-in conditioner use and frequency during pregnancy in relation to gestational age at delivery. In addition, this study collected hair product use data at multiple time points across pregnancy using a validated questionnaire, allowing for exposure assessment at specific prenatal windows that may be etiologically relevant to birth outcomes. Furthermore, our work aimed to assess hair products containing mixtures of EDCs to identify modifiable risk factors, such as specific hair product use patterns, which may contribute to preterm birth rather than focusing on a specific EDC chemical class (e.g., biomarker concentration of phthalates) that may be more difficult to link to product use. This assessment allows for identification of a potentially modifiable risk factor that could contribute to environmental health disparities related to preterm birth.

## Conclusions

Our findings suggest that daily use of hair oils during late pregnancy may be associated with earlier gestational age at delivery. These findings raise key questions about EDC-associated hair product use as a potentially modifiable risk factor for decreased gestational age at delivery, particularly for use over the course of the third trimester.

## Supplementary Information

**Additional file 1:** Supplemental Tables S1-S4.

## Data Availability

The data that support the findings of this study are available on request from the corresponding author, EVP, and PI of the ERGO Study, TJ-T. The data are not publicly available due to institutional restrictions that prohibit the sharing of data containing information that could compromise research participant privacy upon which participant consent was contingent.

## Abbreviations

EDC: Endocrine disrupting chemical
ERGO: Environmental Reproductive and Glucose Outcomes (ERGO) Study
MA: Massachusetts
BIDMC: Beth Israel Deaconess Medical Center
BMI: Body mass index
SD: Standard deviation
CI: Confidence interval
