# Supplementary material for: Endocrine disrupting chemical-associated hair product use during pregnancy and gestational age at delivery: a pilot study
Source: Environ Health. 2021 Jul 28;20:86. doi: 10.1186/s12940-021-00772-5 (PMC8316883; doi:10.1186/s12940-021-00772-5)
Supplement: Supplementary file 1 — Additional file 1: Supplemental Tables S1-S4. [file 12940_2021_772_MOESM1_ESM.docx]

**Supplemental Materials**

**Endocrine Disrupting Chemical-Associated Hair Product Use During Pregnancy and Gestational Age at Delivery: A Pilot Study**

**Authors & Affiliations**

Emma V. Preston^1^*, Victoria Fruh^1^*, Marlee R. Quinn^1^, Michele R. Hacker^2,3^, Blair J. Wylie^2^, Karen O'Brien^2^, Shruthi Mahalingaiah^1,3λ^, Tamarra James-Todd^1,4λ^

*These authors contributed equally to this work

λThese authors share last-authorship

^1^Department of Environmental Health, Harvard T.H. Chan School of Public Health, Boston, Massachusetts, 02115, USA

^2^Department of Obstetrics and Gynecology, Beth Israel Deaconess Medical Center, Harvard Medical School, Boston, Massachusetts, 02115, USA

^3^Department of Obstetrics and Gynecology, Division of Reproductive Endocrinology and Infertility, Massachusetts Gneral Hospital, Boston, MA 02114, USA

^4^Department of Epidemiology, Harvard T.H. Chan School of Public Health, Boston, Massachusetts, 02115, USA

**Corresponding Author**

Emma V. Preston, PhD, MPH

Department of Environmental Health

Harvard T.H. Chan School of Public Health

677 Huntington Ave

Building 1, Room 1305

Boston, MA 02115

Phone: 617-432-1477

Email: epreston@hsph.harvard.edu

**Table S1.** Delivery mode by participant race/ethnicity (n=154)

|  | Non-Hispanic Black | Non-Hispanic White | Other Race |
| --- | --- | --- | --- |
| Delivery Mode | n (%) | n (%) | n (%) |
| Cesarean | 1 (8) | 20 (26) | 16 (25) |
| Vaginal – induced | 3 (25) | 24 (31) | 13 (20) |
| Vaginal – spontaneous | 8 (67) | 33 (43) | 36 (55) |

**Table S2.** Unadjusted Difference in Mean Gestational Age (Days) at Delivery Associated with Frequency of Hair Product Use During Pregnancy (n=154)

| Model Timepoint |  |  | Hair Oil |  | Hair lotion |  | Leave-In  Conditioners |
| --- | --- | --- | --- | --- | --- | --- | --- |
|  | Frequency | n | β (95% CI) | n | β (95% CI) | n | β (95% CI) |
| Visit 1 |  | 134 |  | 136 |  | 134 |  |
|  | Daily | 7 | -4.8 (-14.9, 5.3) | 4 | 3.5 (-9.7, 16.6) | 7 | 6.0 (-4.2, 16.1) |
|  | <Daily | 18 | 1.0 (-5.6, 7.6) | 17 | -3.1 (-9.8, 3.6) | 20 | 1.8 (-4.6, 8.1) |
|  | Never | 109 | 0 (ref) | 115 | 0 (ref) | 107 | 0 (ref) |
| Visit 2 |  | 136 |  | 136 |  | 137 |  |
|  | Daily | 10 | -0.6 (-9.1, 8.0) | 10 | -1.4 (-10.0, 7.2) | 6 | -4.7 (-12.6, 3.1) |
|  | <Daily | 19 | -2.3 (-8.8, 4.2) | 17 | -0.7 (-7.4, 6.2) | 20 | -0.4 (-4.5, 3.8) |
|  | Never | 107 | 0 (ref) | 109 | 0 (ref) | 106 | 0 (ref) |
| Visit 3 |  | 135 |  | 134 |  | 136 |  |
|  | Daily | 14 | -1.1 (-6.2, 4.1) | 9 | 1.0 (-5.3, 7.2) | 5 | -2.3 (-10.5, 5.9) |
|  | <Daily | 12 | -1.1 (-6.6, 4.4) | 12 | -2.1 (-7.6, 3.4) | 20 | -2.1 (-6.5, 2.2) |
|  | Never | 109 | 0 (ref) | 113 | 0 (ref) | 111 | 0 (ref) |
| Visit 4 |  | 129 |  | 127 |  | 129 |  |
|  | Daily | 8 | -7.5 (-13.8, -1.2) | 3 | -2.6 (-12.9, 7.7) | 6 | -2.8 (-10.2, 4.5) |
|  | <Daily | 18 | 0.9 (-3.5, 5.3) | 12 | -2.1 (-7.4, 3.3) | 17 | -0.2 (-4.8, 4.4) |
|  | Never | 103 | 0 (ref) | 112 | 0 (ref) | 106 | 0 (ref) |

**Table S3**. Difference in Mean Gestational Age at Delivery (Days) Associated with Frequency of Hair Oil Use During Pregnancy – Restricted to Spontaneous Vaginal Deliveries (n=77)

| Model  Timepoint |  | n | β (95% CI) |
| --- | --- | --- | --- |
|  | Frequency |  |  |
| Visit 1 |  | 65 |  |
|  | Daily | 4 | -4.9 (-15.1, 5.4) |
|  | <Daily | 6 | 1.0 (-5.7, 7.6) |
|  | Never | 55 | 0 (ref) |
| Visit 2 |  | 70 |  |
|  | Daily | 5 | 0.0 (-8.7, 8.8) |
|  | <Daily | 7 | -2.3 (-8.9, 4.3) |
|  | Never | 58 | 0 (ref) |
| Visit 3 |  | 68 |  |
|  | Daily | 7 | -1.1 (-6.4, 4.2) |
|  | <Daily | 5 | -1.3 (-6.9, 4.3) |
|  | Never | 56 | 0 (ref) |
| Visit 4 |  | 69 |  |
|  | Daily | 5 | -7.9 (-14.3, -1.5) |
|  | <Daily | 11 | 0.9 (-3.5, 5.3) |
|  | Never | 53 | 0 (ref) |

^a^Adjusted for maternal age at enrollment (years)

**Table S4.** Difference in Mean Gestational Age at Delivery (Days) Associated with Frequency of Hair Oil Use During Pregnancy – Sensitivity Analysis, Restricted to Deliveries prior to March 1^st^, 2020, pre-COVID-19 shutdown in MA

| Model  Timepoint |  | n | Crude | Adjusted^a^ |
| --- | --- | --- | --- | --- |
|  | Frequency |  | β (95% CI) | β (95% CI) |
| Visit 1 |  | 101 |  |  |
|  | Daily | 7 | -5.1 (-16.0, 5.9) | -4.7 (-15.9, 6.5) |
|  | <Daily | 11 | -0.4 (-9.3, 8.6) | -0.3 (-9.3, 8.8) |
|  | Never | 83 | 0 (ref) | 0 (ref) |
| Visit 2 |  | 100 |  |  |
|  | Daily | 9 | -1.0 (-10.8, 8.9) | -0.3 (-10.5, 10.0) |
|  | <Daily | 13 | -3.6 (-12.0, 4.8) | -3.3 (-11.9, 5.3) |
|  | Never | 78 | 0 (ref) | 0 (ref) |
| Visit 3 |  | 101 |  |  |
|  | Daily | 10 | -1.8 (-7.8, 4.3) | -1.5 (-7.9, 5.0) |
|  | <Daily | 8 | -2.1 (-8.7, 4.6) | -1.8 (-8.6, 5.0) |
|  | Never | 83 | 0 (ref) | 0 (ref) |
| Visit 4 |  | 100 |  |  |
|  | Daily | 7 | -8.8 (-15.4, -2.2) | -9.7 (-16.5, -2.8) |
|  | <Daily | 14 | 0.7 (-4.1, 5.5) | 0.1 (-4.5, 5.5) |
|  | Never | 82 | 0 (ref) | 0 (ref) |

^a^Adjusted for maternal age at enrollment (years) and delivery mode (cesarean, induced vaginal, spontaneous vaginal)
